# Polylactic Acid-Based Patterned Matrixes for Site-Specific Delivery of Neuropeptides On-Demand: Functional NGF Effects on Human Neuronal Cells

**DOI:** 10.3389/fbioe.2020.00497

**Published:** 2020-06-12

**Authors:** Olga A. Sindeeva, Olga Kopach, Maxim A. Kurochkin, Andrei Sapelkin, David J. Gould, Dmitri A. Rusakov, Gleb B. Sukhorukov

**Affiliations:** ^1^Center for Neurobiology and Brain Restoration, Skolkovo Institute of Science and Technology, Moscow, Russia; ^2^Remote Controlled Theranostic Systems Lab, Department of Nanotechnology, Educational and Research Institute of Nanostructures and Biosystems, Saratov State University, Saratov, Russia; ^3^UCL Queen Square Institute of Neurology, University College London, London, United Kingdom; ^4^Center for Photonics and Quantum Materials, Skolkovo Institute of Science and Technology, Moscow, Russia; ^5^School of Physics and Astronomy, Queen Mary University of London, London, United Kingdom; ^6^Biochemical Pharmacology, William Harvey Research Institute, Queen Mary University of London, London, United Kingdom; ^7^School of Engineering and Material Science, Queen Mary University of London, London, United Kingdom; ^8^Center of Biomedical Engineering, I.M. Sechenov First Moscow State Medical University, Moscow, Russia

**Keywords:** polylactic acid (PLA), patterned microchamber array, drug delivery system, NGF, human N2A cells

## Abstract

The patterned microchamber arrays based on biocompatible polymers are a versatile cargo delivery system for drug storage and site-/time-specific drug release on demand. However, functional evidence of their action on nerve cells, in particular their potential for enabling patterned neuronal morphogenesis, remains unclear. Recently, we have established that the polylactic acid (PLA)-based microchamber arrays are biocompatible with human cells of neuronal phenotype and provide safe loading for hydrophilic substances of low molecular weight, with successive site-specific cargo release on-demand to trigger local cell responses. Here, we load the nerve growth factor (NGF) inside microchambers and grow N2A cells on the surface of patterned microchamber arrays. We find that the neurite outgrowth in local N2A cells can be preferentially directed towards opened microchambers (upon-specific NGF release). These observations suggest the PLA-microchambers can be an efficient drug delivery system for the site-specific delivery of neuropeptides on-demand, potentially suitable for the migratory or axonal guidance of human nerve cells.

## Introduction

Micro- and nanostructured matrices have prompted new lines of study focusing on cell behavior (adhesion, proliferation, morphology, alignment, migration, gene expression, and even differentiation) and tissue engineering (Sousa et al., 2019). Photolithography and electroplating techniques enable creation of templates with the choice of different geometries. Such templates can be used either as independent matrices for growing cells, or as templates for the reusable synthesis of patterned films composed of polymers, proteins, and colloids, with nanoscale fidelity.

The current patterning techniques to control nerve cell morphogenesis and function include the NeuroArray device designed for the patterned growing of nerve cells (Li et al., 2014), graphene oxide-based hybrid patterns for guiding axonal growth (Min et al., 2017), microcontact-printed polymeric substrates for directed neuronal regeneration (Schmalenberg and Uhrich, 2005), micropatterned polymer brushes for cell directionality (Pardo-Figuerez et al., 2018), and others. The ultimate goal of these techniques has been, however, to modify neuronal growth and cell proliferation, with no attempts to stimulating activity of individual cells. A recently developed patterned microchamber array (MCA) (Kiryukhin et al., 2018) is, therefore, of particular importance: it provides a reservoir matrix consisting of microchambers (microcontainers). Such a matrix can be made of various synthetic and biocompatible polymers using the layer-by-layer method (Ermakov et al., 2019). MCA could be composed of a variety of hydrophobic polymers, for instance, polylactic acid (PLA) (Zykova et al., 2019), poly(lactic-co-glycolic acid) (Sindeeva et al., 2018a), etc., with various inclusions inserted into the shell of microchambers, such as gold nanoparticles or the aggregates of carbon dots (Sindeeva et al., 2018b; 2019; Kurochkin et al., 2020). Having biologically active substances inside microchambers allows spatially and temporally modulated control of cell function, not only due to the periodic structure of the material (Norman and Desai, 2006; Bettinger et al., 2009; Ge et al., 2015; Sousa et al., 2019) but also due to the encapsulated cargo release (Kopach et al., 2019). A wide range of biocompatible polymers for the MCA synthesis enables control of the cargo release rate. For example, it was shown that the PLGA-based microchambers provided relatively slow, continuous release of adrenaline hydrochloride from the first day after entering the aqueous environment (Sindeeva et al., 2018a), which has a clear advantage in many clinical applications. The release of a significant quantity of cargo can be induced by ultrasound as a result of the simultaneous opening of many microchambers (Sindeeva et al., 2018a), or otherwise individual chambers can be opened by optical laser targeting (Sindeeva et al., 2018b; Kopach et al., 2019; Kurochkin et al., 2020).

Notwithstanding the advantages of MCA as a system for targeted delivery of drugs and biologically active substances, its applications in human cells remain poorly understood; this precludes perspective clinical use of these systems. This is mainly because the methods of encapsulation, the duration of storage, the release rate of the substance depend not only on the shell material (Lee and Yeo, 2015) and container geometry (Macha et al., 2019), but also on the cargo's chemical and physical properties (Albinali et al., 2019), which vary widely. Earlier, we have demonstrated that PLA-based MCA are fully biocompatible with human cells of neuronal phenotype (Kopach et al., 2019). Furthermore, we showed a site-specific cellular response to the release of compounds of low molecular weight from individual microchambers: doxycycline for enhancing biosynthesis of green fluorescent protein in individual C2C12 cells (Gai et al., 2018) and the excitatory neurotransmitter glutamate for activation of N2A cells (Kopach et al., 2019). These observations have validated MCA as an effective delivery system for modifying cell activity on demand.

The site- and time-specific delivery of neuropeptides is another important step in managing nerve cell growth, including directed neurite outgrowth that has strong potential in neuroregenerative medicine. Here, we demonstrate the feasibility of modulating neuronal cell function through loading and site-specific release of the nerve growth factor (NGF), a neuropeptide which is primarily involved in the regulation of neurotrophic activities, growth, and proliferation of nerve cells (Levi-Montalcini, 1987). Since NGF is a neuropeptide, it is particularly sensitive to the small temperature fluctuations characteristic of laser exposure. The laser-triggered NGF release from MCA to the targeted N2A cells growing on the MCA surface was therefore performed using focused near-infrared (NIR) laser light. The advantage of using NIR laser rests with the minimal light absorption of biological tissues within the 650–975 nm spectral range, which minimizes photothermal effects on cells. The site-specific localization of the laser-triggered photothermal influence on the chamber wall was enabled by the inclusion of gold nanoparticles (GNPs) in the shells of microchambers as highly photo-absorbing agents (Wijaya et al., 2009; Agarwal et al., 2011). GNPs is a safe (Sperling et al., 2008; Boisselier and Astruc, 2009), well-established, and widely used thermosensitive material for polymer carrier opening *in vitro* and *in vivo* (Radt et al., 2004; Skirtach et al., 2005; Boisselier and Astruc, 2009; Singh, 2010; Kunzmann et al., 2011).

## Materials and Methods

### Materials

For MCA synthesis, biopolymer PLA (3 mm granules, molecular weight 60,000), chloroform, and NGF-β (molecular weight 13,5 kDa) were purchased from Sigma-Aldrich (UK). The Poly(dimethylsiloxane) (PDMS) kit (Sylgard 184) was purchased from Dow-Corning (Midland, USA).

For gold nanorods (GNRs) synthesis, cetyltrimethylammonium bromide (CTAB, >98.0%), hydrochloric acid (HCl, 37 wt% in water), L-ascorbic acid (>99.9%), and sodium borohydride (NaBH_4_, 99%) were purchased from Sigma-Aldrich (UK). Hydrogen tetrachloroaurate trihydrate (HAuCl_4_·3H_2_O) and silver nitrate (AgNO_3_, >99%) were purchased from Alfa Aesar.

### Synthesis of GNRs

GNRs were fabricated by the modified seed-mediated method (Nikoobakht and El-Sayed, 2003; Khlebtsov et al., 2011). At the first step, the seed solution was obtained by mixing 250 μL of 10 mM HAuCl_4_ and 10 mL of 0,1 M CTAB. The ice-cold 10 mM NaBH_4_ was added to the mixture in the volume of 1 mL. At the second step, 10 mL of the seed solution were mixed with 900 μL of 0.1 M CTAB, 20 mL of 4 mM AgNO_3_, 50 mL of 10 mM HAuCl_4_, 10 mL of 1 M HCl, and 10 mL of 0.1 M ascorbic acid for preparing GNRs. Then nanorods were centrifuged at 12,000 g for 60 min. The pellet was re-suspended in deionized water. The final solution was containing about 1012 GNRs per mL; their average width was 11 ± 3 nm and length was 40 ± 6 nm. The axial ratio was ~3.8, according the longitudinal resonance was ~790 nm.

### Fabrication of PLA-Based MCA

**Figure 1:**
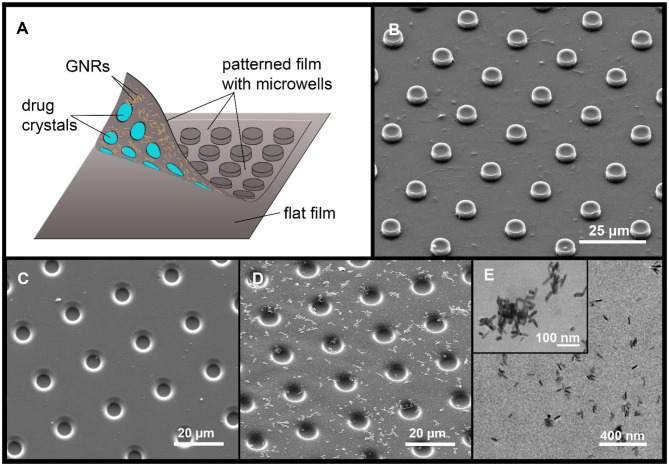
A diagram of the MCA, with a drug cargo and GNRs **(A)**. Typical SEM image of PLA-based MCA with GNRs **(B)**. SEM images of PLA patterned film, without **(C)** and with GNR aggregates **(D)**. The arrangement of GNR aggregates (**E**, TEM image).

The silicon master was previously made at Shenzhen Semiconductor (Shenzhen, China) using photolithography for MCA synthesis. The pattern on silicon master was represented by a plate with 185,000 cylinders equidistant from each other (diameter 10 μm, height 4 μm, and distance from center to center 20 μm). For the synthesis of patterned films, the PDMS stamp was made as a reverse impression from a silicone master from a mixture of the prepolymer and curing agent (10:1 ratio). The mixture was degassed for 30 min in a vacuum and consolidated (at 70°C for 3 h). After this, the PDMS master was cut out and separated from the silicon master. The shell of the PLA-based MCA was made by sealing (printing) of two films: the patterned and the flat ones (2 kg cm^−2^, 15 s, at 55°C) (Figure 1A). For the synthesis of the patterned film, the PDMS stamp with microwells was dip-coated for 5 s into the 1 wt% PLA chloroform solution; for obtaining the flat PLA microfilm, the same procedure was made with a cover glass. After printing, the PDMS stamp was removed and the MCA was located on cover glass.

The patterned film was covered with GNRs before printing by scattering GNRs on the inner surface of the PLA film by sedimentation (Sindeeva et al., 2018b). For precipitation and sedimentation of GNRs, 200 μL of 0.5 M NaCl solution was added to 200 μL of the nanoparticles solution to enhance aggregation (Madzharova et al., 2018). The resulting solution was centrifuged at 10,000 rpm, and supernatant was removed. Next, GNRs were resuspended in 200 μL of deionized water. After that procedure, aggregates of GNRs started to adsorb on a hydrophobic PLA surface. Aggregates in comparison with non-aggregated particles have a larger size and mass which led to amplification of sedimentation rate (Midelet et al., 2017). As a result, aggregates of gold nanoparticles were visualized with an optical microscope, as well as with scanning electron microscopy (SEM) and transmission electron microscopy (TEM).

### NGF Loading Into Microchambers; NGF Leak Test

**Figure 2:**
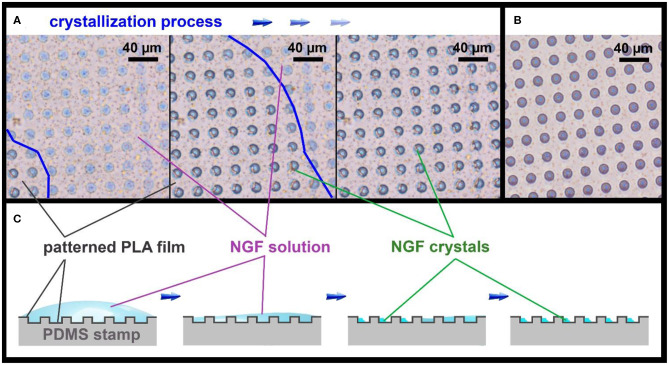
Optical images of the patterned PLA film **(A)**, showing the NGF crystallization process, and an empty patterned film **(B)**, bright-field microscopy in phase contrast mode. The border of NGF solution drop is marked with a blue line. Schematic illustration of the NGF crystallization process on the patterned PLA film **(C)**.

**Figure 3:**
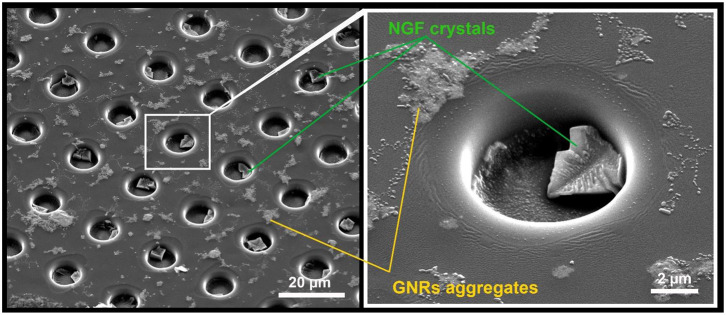
SEM images of NGF crystals inside the microwells on patterned PLA film.

NGF loading was carried out by applying 10 μL of an aqueous solution (10 μg/mL) on the inner surface of the patterned film after the deposition of GNRs. For homogeneous loading, the solution was evenly distributed over the entire film surface and allowed to completely dry (Figures 2A–C and Supplementary Movie 1). To confirm homogeneous loading of the microwells, NGF crystals were visualized inside the fabricated microchambers with SEM (Figure 3).

To test whether spontaneous NGF release from the microchambers could occur after sealing, the fabricated MCA were placed in Dulbecco's phosphate-buffered saline (DPBS) at 37°C (95% O_2_ and 5% CO_2_) to represent a relatively physiological environment. The amount of NGF was tested in DPBS at different time-points for up to 3 days using the Brilliant Blue G reagent and spectrophotometry.

### Laser-Induced Opening of Individual PLA-Based Microchambers

**Figure 4:**
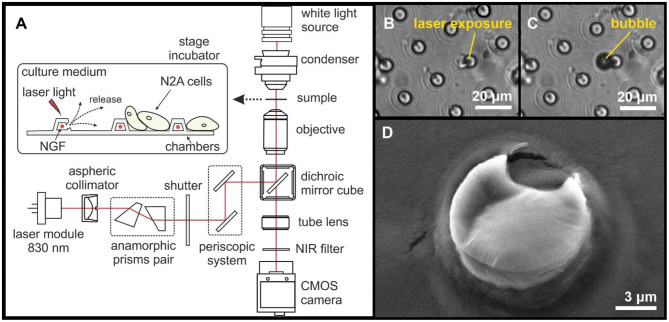
A diagram illustrating the experimental design and the laser-induced opening of MCA with NGF loaded inside **(A)**. Typical images of microchambers before **(B)** and after **(C)** laser exposure (bright-field microscopy). SEM image of an opened microchamber **(D)**.

The NIR lasers are widely used for the opening of targeted drug delivery systems as they are associated with good penetration ability in tissue without damaging living cells. Laser-induced opening of individual PLA microchambers with the N2A cells growing on the MCA surface was performed using a home-made system. The in-house-made system was based on an inverted microscope (Olympus ix71, Japan), into the optical path of which we integrated a continuous-wave NIR laser module (LD830-MA1W, 830 nm, maximum optical power 1W, Thorlabs Inc., USA) with adjustable output power, to enable photo-thermal activation of selected microchambers (Figure 4A). First, NIR laser light was collimated by an aspheric lens and was 3x expanded by an anamorphic prism pair. Next, laser light was directed into the microscope infinity port by a two-mirror periscope. Then, the laser light was directed by an infrared short-pass dichroic mirror (DMSP805, 805 nm cutoff wavelength, Thorlabs Inc., USA) into an exit pupil of an infinity-corrected objective lens LCAch 20x/0.4 PhC (Olympus, Japan) and focused by the objective into a 1 μm spot on the surface of a selected microchamber, at a power of 15 mW over 0.5 s. The laser light irradiation exposure time was controlled by a mechanical shutter. The optical system has been tuned for the confocality between the transmitted light and the NIR channel to control the NIR channel focus by the visible-light focus. Thus, we routinely focused the NIR laser on the microchamber base to minimize damage, if any, to NGF crystals which tend to accumulate toward the top (Figure 3). The N2A cell reaction to triggered NGF release was recorded using a monochrome CMOS sensor (DCC3260M, Thorlabs Inc., USA) with an infrared filter.

### SEM and TEM Techniques

To visualize MCA morphology at different steps through the fabrication procedure (payload, sealing) and after opening microchamber(s), SEM was used to ensure appropriate samples (FEI Quanta ESEM, electron microscope, FEI, Hillsboro, USA). SEM was carried out using an accelerating voltage of 10 kV, a spot size of 3.5, and a working distance of ~10 mm.

TEM images of the MCA with GNRs were obtained using a Jeol 2100 microscope (Tokyo, Japan). GNRs diameters and lengths were evaluated from digitized TEM images (Grapher 8, Golden Software, Inc.) of about 500 GNRs.

### Human N2A Cell Culture

To test functional effects of laser-triggered release of NGF from PLA-microchambers, we used human N2A cells. The cell line was maintained as we have recently described in detail (Kopach et al., 2019). Briefly, N2A cells were cultured in Dulbecco's modified Eagle medium (DMEM, Invitrogen, Carlsbad, CA, USA), supplemented with 2 mM L-glutamine and 10% fetal bovine serum, 2% penicillin-streptomycin, and 1% non-essential amino acids at 37 °C (5% CO_2_). After harvesting, cells were washed out and plated on a surface of PLA-based MCA, pre-treated with UV light for at least 2 h in advance. For cell differentiation to neuronal phenotype, the culturing medium was low serum (2%) DMEM. We used N2A cells at earlier passages only (before cells pass passage 20). Microscopic images of differentiating N2A cells on the fabricated MCA were collected before microchamber opening and afterward at various time-points. For the time-lapse imaging, a MCA with differentiating N2A cells on its surface was placed in a microscope cage incubator (5 cm^2^ Petri dish) to maintain experimental conditions favorable for live-cell imaging (37°C, 5% CO_2_). Images were acquired every 10 min for up to 60 h total.

In separate experiments, N2A cells were plated on glass coverslips placed into a 8 × 8 wells-plate. Experimental groups consisted of the cells of the same passage grown on glass in culture medium without NGF or NGF supplemented at the concentration of 10 or 100 ng/mL. There were at least four independent samples tested for each experimental group.

### Assessment of Neurite Length; Cell Density Analysis

Neurite outgrowth by N2A cells was assessed by measuring the neurite length in different experimental conditions, using a NeuronJ, a plugin of ImageJ software (NIH, Bethesda, USA). Neurites were traced in individual cells manually, using variable digital zooming. Analyses were performed in the cell culture field of view, across multiple areas selected in a pseudo-random manner.

The N2A cell density was analyzed by counting cell bodies on the surface of the fabricated array, within the area of interest (close to the opened microchambers). Cell density was estimated as the number of viable cells per mm^2^ over the selected period of time-lapse recording, as indicated.

### Statistical Analysis

Data are presented as mean ± standard error of the mean, with n referring to the number of neurites measured for their length, for each experimental group. To determine the statistical difference between experimental groups, two-tailed unpaired Student's *t*-test was used. A *p* < 0.05 was considered as an indicator of the statistically significant difference.

## Results and Discussion

### PLA-Based MCA With Gold Nanoparticles: Fabrication and Characterization

We created MCA by printing both flat and patterned films (Figure 1A). The thickness of the finished film was 0.8–1.0 μm. GNPs (as a classical method) were included in the MCA composition before printing, to enable controlled opening of microchambers with laser light (Singh, 2010; Kunzmann et al., 2011).

For surface modification, an in-advance concentrated water solution with GNRs was prepared (200 mg/mL). Two hundred microliter of this solution were placed on the inner surface of the patterned microfilm with microwells, for 3 h. During this time, the patterned film was horizontally oriented, after which the drop was removed using a micropipette. The entire surface of the patterned film was covered with GNR aggregates, which were clearly visible under an optical microscope. Figure 1 shows an SEM image (D) and TEM image (E) of the GNR aggregates location. GNRs content in the patterned film was 0.47 pg/μm^2^, as estimated from the absorption spectrum change in the solution, before and after deposition of aggregates.

### NGF Loading

Microchambers were filled by applying 10 μL of the NGF solution (concentration 10 μg/mL) on the patterned PLA film surface (8.5 × 8.5 mm, 185000 microwells), before printing it on a flat film. Although the PLA film has hydrophobic properties (Alakrach et al., 2018), the NGF solution uniformly wetted the patterned surface due to the low surface tension. The drying of the NGF solution occurred evenly over the entire film surface, with a gradual decrease of the solution drop thickness (Figure 2A). When the water layer thickness reached a critical point, the rapid formation of crystals in the wells began over the entire surface (Figures 2A,C). The crystallization process could be clearly observed in a light microscope in real time (Supplementary Movie 1).

The images obtained using SEM confirmed the uniformity of filling the microwells with crystals, and the absence of NGF between them (Figure 3).

In general, the amount of NGF was 100 ng per sample (8.5 × 8.5 mm) and 0.54 pg per microchamber. This amount was calculated theoretically, by taking into account the total amount of substance deposited on the patterned film surface, and the number of microwells. This amount of NGF was highest possible to achieve reliable loading into the microwells of pre-designed configuration (diameter of 10 μm, height 4 μm). Further concentration increases led to the formation of crystals across the entire film surface (outside of the microwells) that prevented tight and reliable sealing of microchambers by the two films.

In order to increase the capability of NGF payload, larger microchambers can be used. At the same time, the use of larger (especially taller) microchambers may cause difficulties for cells to freely move, since the surface topography was found to have substantial effects on the cells behavior in different cell types (Norman and Desai, 2006; Bettinger et al., 2009; Ge et al., 2015; Sousa et al., 2019).

Controllable microchamber permeability is an important issue, which we addressed in some detail in our previous work for the PLA-based MCA loaded with low molecular weight compounds (Kopach et al., 2019). To examine potential leakage of NGF from PLA-microchambers, we next carried out testing of the fabricated MCA loaded with NGF in DPBS (at 37°C, 95% O_2_ and 5% CO_2_) over time and analyzed the amount of NGF in DPBS at different time-points. The amount of NGF that could leak out did not exceed 10–12% after 3 days of incubation in a mimicked physiological microenvironment (Supplementary Figure 1). Notably, that level does not exceed sensitivity of the assay. It should be also noted that we detected no leak of cargo payload in our previous study in which the PLA-microchambers contained the excitatory neurotransmitter glutamate over at least 1 week (Kopach et al., 2019).

Clearly, the release and dissolution kinetics of signaling molecules in aqueous physiological solutions (the point of interest here) could vary widely across molecular species. Furthermore, control over this process could be an important issue in the experimental design. In our case, it has been difficult to detect small amounts of NGF in the medium, so that is why we focused on documenting its neurophysiological effects.

### Individual Microchamber Opening Using NIR Laser

The NIR laser light (15 mW for 0.5 s) was focused only onto a ~1 μm spot over an individual microchamber (i.e., on the microchamber base) to ensure the most local effect on the GNR aggregates in the microchamber wall. The exposure to laser light at 830 nm (Figure 4B) was accompanied by the appearance of a small gas bubble (Figure 4C) and by structural changes of the microchamber surface (Figure 4D). The microscopic bubble formation is associated with the liquid boiling on GNRs surface as a result of energy absorption and fast plasma formation occurring after liquid evaporation and subsequent vapor expansion, which are accompanied by a shock wave (Lauterborn and Ebeling, 1977; Baghdassarian et al., 1999; Link et al., 2000; Link and El-Sayed, 2001).

NIR lasers are used for heating up GNRs in the polymer shell because GNRs efficiently absorb laser energy (Gordel et al., 2014). The heating of light absorber agents such as GNRs by laser irradiation leads to the rapid melting of the carrier walls and subsequent cargo release (Radt et al., 2004; Skirtach et al., 2005, 2007; Singh, 2010). Although we did not measure the dynamics of local temperature during the laser-induced opening, it is known that laser irradiation of metallic plasmonic nanoparticles causes a very local heat increase, especially for isolated GNRs. In such cases, the temperature drops exponentially around plasmonic particles having a negligible impact on the environment (Govorov and Richardson, 2007), which was demonstrated in multilayer capsules with embedded GNR where the elevation of heat is within a one-micron spot (Skirtach et al., 2008). This has been presently confirmed to have no impact on live cells over a micron distance away from the laser exposure zone (Gai et al., 2018; Kopach et al., 2019). Again, we note that light absorption of an aqueous solution peaks at ~970 nm. At 830 nm used here, it drops ~20 times: in our case, the bulk of the energy is absorbed by nanoparticles and released as mechanical decomposition rather than heat. Besides, the structural PLA changes were found to take place from above 50°C (Zhou et al., 2015), with the reported PLA glass-melting transition point near 55–60°C (Marek and Veney, 2016). We ensured that no neighboring cells were affected by the NIR laser light during the microchamber opening as evidenced by the images shown.

### Directed Neurite Outgrowth by Local N2A Cells Towards the Opened Microchambers With NGF Payload Inside

**Figure 5:**
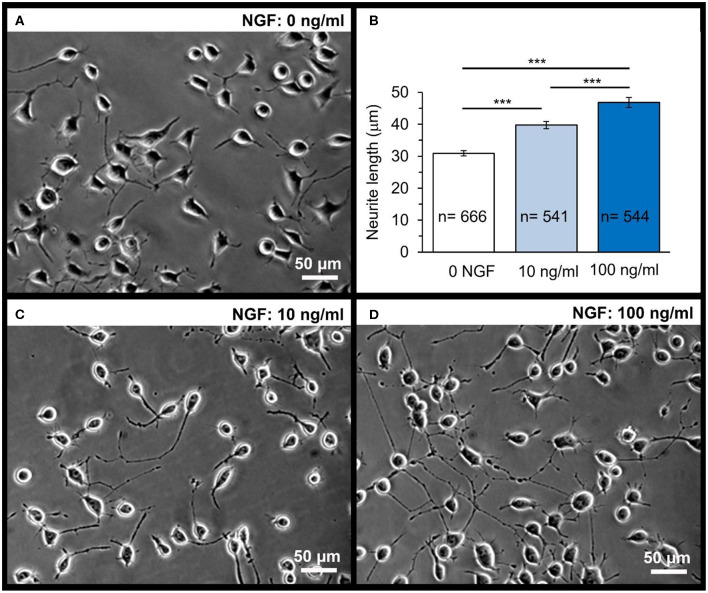
Dose-dependent effect of NGF on the neurite outgrowth in N2A cells on glass coverslips. A snapshot of differentiating N2A cells after 1 day of cell growth on glass **(A)**. Statistical summary of the neurite length in N2A cells grown without or with NGF supplemented to culture medium at the concentration of 10 or 100 ng/mL. Numbers of neurites measured for their length are indicated; at least four independent samples (coverslips) were tested for each group. ***P < 0.001 (two-tailed, unpaired *t*-test). **(B)** Representative images of differentiating N2A cells after 1 day of cell growth with NGF at different concentrations: 10 ng/mL **(C)** or 100 ng/mL **(D)**.

Next, we sought to test functional effects of NGF following the laser-triggered opening of microchambers. We utilized the human N2A cell line, a cell type providing rapid cell growth and differentiation to neuronal phenotype of human origin, as shown previously (Kopach et al., 2019). As expected, differentiating N2A cells developed typical axon-like processes and numerous neurites 1 d post-plating (Figure 5A), which could extend up to 50 μm in length, with morphogenesis progressing during cell growth.

First, we evaluated the NGF-induced effects on differentiating N2A cells grown on glass (control group). Since NGF is a highly potent neuropeptide acting in the ng/mL concentration range, we supplemented NGF to culture medium at the concentration of 10 and 100 ng/mL. There was a clear, dose-dependent effect of NGF on the neurite outgrowth by N2A cells observed after 1 day of cell differentiation with NGF (Figures 5C,D). The neurite length was on average ~31.1 μm in control (0 NGF, *n* = 666 neurites), but ~40.3 μm in the presence of 10 ng/mL NGF (*n* = 541 neurites; *p* < 0.0001) and ~46.8 μm with 100 ng/mL NGF (*n* = 544 neurites, *p* < 0.0001; Figure 5B) for N2A cells of the same passage.

**Figure 6:**
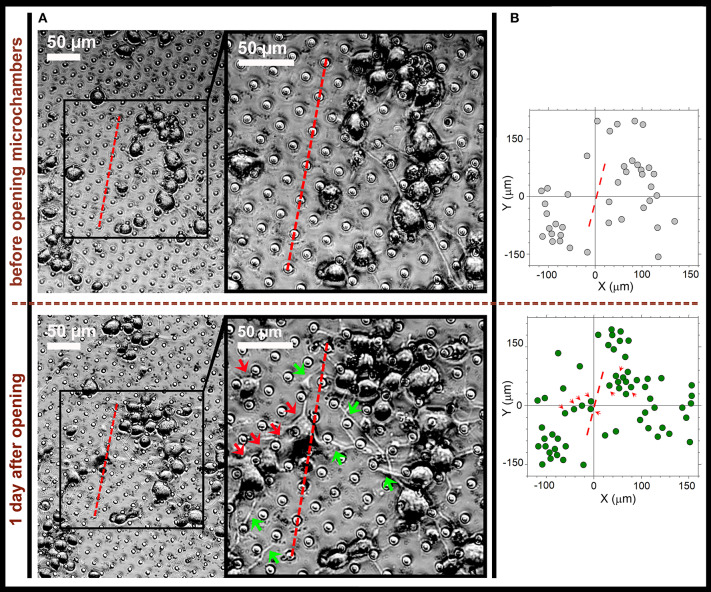
Directed neurite outgrowth by local N2A cells and cell migration toward the laser-opened microchambers with NGF payload inside. **(A)** Representative snapshots of differentiating N2A cells growing on the surface of PLA-based MCA with NGF payload inside microchambers before microchamber opening (upper row) and 1 day after (lower row). Red dotted line, a line segment trajectory for optical targeting microchambers (7 microchambers opened). Red arrows, directed migration of individual cells from their original positions; green arrows, cell neurites directed toward the opened microchambers (NGF release). **(B)** Cell tracking diagrams depicting individual N2A cell positions before laser-triggered microchamber opening (top) and 1 day after (bottom). Note directed migration of local cells (red arrows) from their original positions toward the opened microchambers. Data are representative of images on **(A)**.

**Figure 7:**
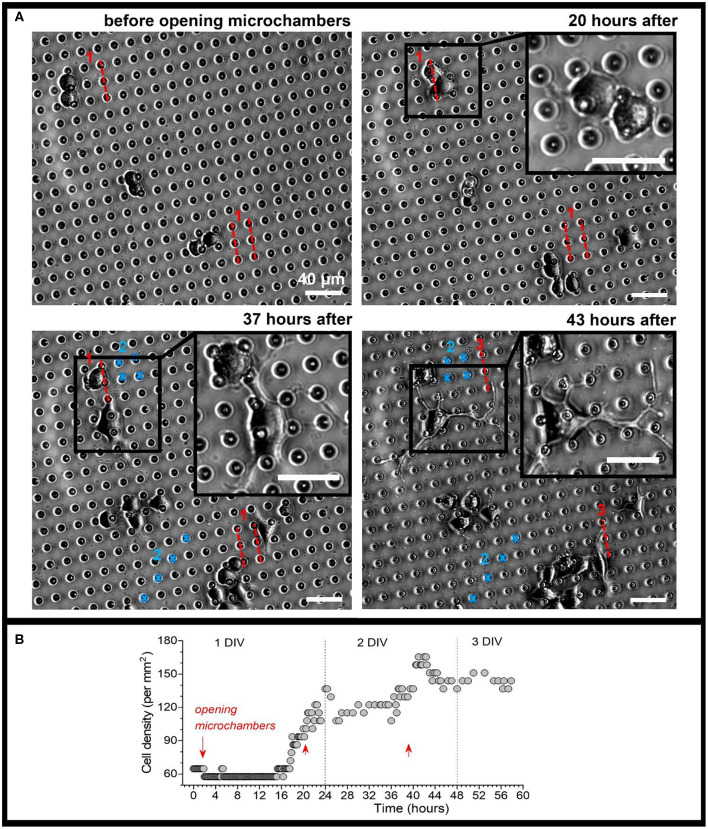
Monitoring morphogenesis of differentiating N2A cells upon triggered, site-specific NGF release from PLA-microchambers. **(A)** Representative snapshots of differentiating N2A cells on the surface of MCA with NGF-loaded microchambers before and following laser-triggered microchamber opening at various time-points. Images taken from the same area of interest; red dotted lines and blue marks, trajectories for optical targeting (the sequence consists of varied trajectory for opening 3 times, ~20 h apart). Scale bars, 40 μm. **(B)** Time-course of cell density changes within the targeted area during the time-lapse imaging (~60 h total) before and following triggered NGF release from PLA-microchambers, shown on **(A)**. Red arrows, time of laser-triggered opening.

Next, we placed N2A cells on the surface of the fabricated MCA with NGF payload inside microchambers, and grew the cells on MCA. The cells showed no signs of toxic damage during their growth on the top of the fabricated MCA, thus confirming biocompatibility for this type of carriers. We monitored uninterrupted growth of differentiating N2A cells growing on the top of arrays, and away from the arrays, over at least 3 days, with no detectable location-specific deterioration of any kind. This is consistent with the previously reported biocompatibility of PLA as the constituent material in our recent study involving the same cell type and a variety of microchamber cargo loads (Kopach et al., 2019). We monitored N2A cells before and after laser-triggered opening of microchambers, throughout the area of interest for several days. We could observe that, after microchamber opening, the cell density increased within the targeted area (Figures 6A,B) and that local cells extended their neurites towards the opened microchambers (Figure 6A, Supplementary Figure 2). These effects were observed across 6 independent experiments (fabricated MCA/cell preparations), at the day 1 or 2 after opening. The effect was observed regardless of the trajectory applied for microchamber opening: a line segment (Figure 6A, Supplementary Figure 2), or a sequence of line followed by rectangular or square shape (Figure 7A, Supplementary Movies 2, 3). These physiological effects further demonstrate that the heat-induced PLA melting required to open individual microchambers for site-specific NGF release was highly localized, leaving the integrity of most NGF molecules intact.

Finally, we carried out time-lapse recording of differentiating N2A cells before and after opening microchambers with NGF payload, by collecting images from the area of interest every 10 min, starting soon after plating N2A cells on the PLA arrays, for up to 3 days in total (Figure 7, Supplementary Movie 2). We detected an increased cell density within the targeted area, with a sharp rise in response to each sequence of microchamber opening (3 times, ~20 h apart) (Figure 7B). On a finer scale, cells growing in close proximity to the opened microchambers directed their neurites towards the sites of NGF release from opened microchambers (Figure 7, images on an expanded scale; Supplementary Movie 3). These results demonstrate a directed neurite outgrowth triggered by the site-targeted cargo release from PLA-microchambers on demand. Moreover, the physiological effects observed following the light-triggered NGF release after ~20 h confirm robust preservation of functional NGF inside microchambers over an extended time. This enables prolonged load storage and selective microchamber opening at a required time point, at a selected microscopic location.

The effects of NGF observed here in human N2A cells of neuronal phenotype are similar to those attributed to NGF across the literature when tested in cell lines and primary neuronal cultures (Craig and Banker, 1994; Jareb and Banker, 1997; Brann et al., 1999; Secondo et al., 2015; Selvaraj et al., 2015). This suggests a potential, in using this type of carrier matrixes, for functional modulation of individual cell activity in response to triggered release of neuropeptides.

## Conclusion

The patterned PLA-based MCA are a versatile drug delivery system for site-specific, geometrically constrained cargo release on demand. Here, we confirm that the PLA-based matrix is fully biocompatible with human-derived cells, which is particularly important for highly sensitive cells of neuronal phenotype. Microchambers appear to provide safe loading for hydrophilic peptides and, because of the presence of light-absorbing gold nanoparticles in the container shell, enable laser-sensitive, site-specific cargo release on demand. Optical targeting of microchambers for drug release has triggered functional cell responses locally. Importantly, N2A cells demonstrate enhanced neurite outgrowth toward individual microchambers releasing NGF. The PLA-based MCA are therefore a potentially suitable platform for site-specific targeting of neuronal cells of human origin.

## Data Availability Statement

The raw data supporting the conclusions of this article will be made available by the authors, without undue reservation, to any qualified researcher.

## Author Contributions

OS, OK, MK, AS, DR, and GS: contributed conception and design of the study. GS, DR, OS, OK, and MK: experiment design and manuscript writing. OS, OK, and MK: conducting experiments. OK: cells state statistical analysis. MK and AS: design and development of optical system for chambers activation. DG: discussions. DR and GS: project supervision. All authors contributed to manuscript writing and revisions, they have approved the submitted version.

## Conflict of Interest

The authors declare that the research was conducted in the absence of any commercial or financial relationships that could be construed as a potential conflict of interest.
