# Supplementary material for: Polylactic Acid-Based Patterned Matrixes for Site-Specific Delivery of Neuropeptides On-Demand: Functional NGF Effects on Human Neuronal Cells
Source: Front Bioeng Biotechnol. 2020 Jun 12;8:497. doi: 10.3389/fbioe.2020.00497 (PMC7304324; doi:10.3389/fbioe.2020.00497)
Supplement: Supplementary file 4 [file Data_Sheet_1.docx]

**Supplementary Materials to the paper**

“Polylactic acid-based patterned matrixes for site-specific delivery of neuropeptides on-demand: functional NGF effects on human neuronal cells”

by

**Olga A. Sindeeva^1,2^**^†^**, Olga Kopach^3†^, Maxim A. Kurochkin^4^, Andrei Sapelkin^5^, David J. Gould^6^, Dmitri A. Rusakov^3*^ and Gleb B. Sukhorukov^1,7,8*^**

**Supplementary Methods**

**Imaging processing for tracking the N2A cell activity presented on video**

The time-lapse images were processed for the changes in activity of N2A cells within the same field of view before and following laser-triggered opening of PLA-microchambers with NGF payload inside. For this, the method of particle image velocimetry was used, which is based on the calculation of the correlation function for the corresponding segments of two consecutive images. Maximum of the correlation characterizes the displacement of image elements relative to the center of the selected interrogation region (IR) with the size of 40x40 pixels, as described by the equation:

$$F_{k}\left( m,n \right)=\sum_{j=1}^{q} \sum_{i=1}^{p} f_{k}\left( i,j \right)g_{k}\left( i+m,j+n \right),$$

where $F_{k}\left( m,n \right)$ is the correlation function, *f_k_(i,j)* and *g_k_(i,j)* are distributions of the pixels intensity of the corresponding IRs with sizes of p x q pixels of two consecutive frames. The velocity of cell movement was calculated by multiplying the displacement of image elements and the time between two frames; this was represented in a pseudo-color manner where blue is minimal movement and red is maximal.


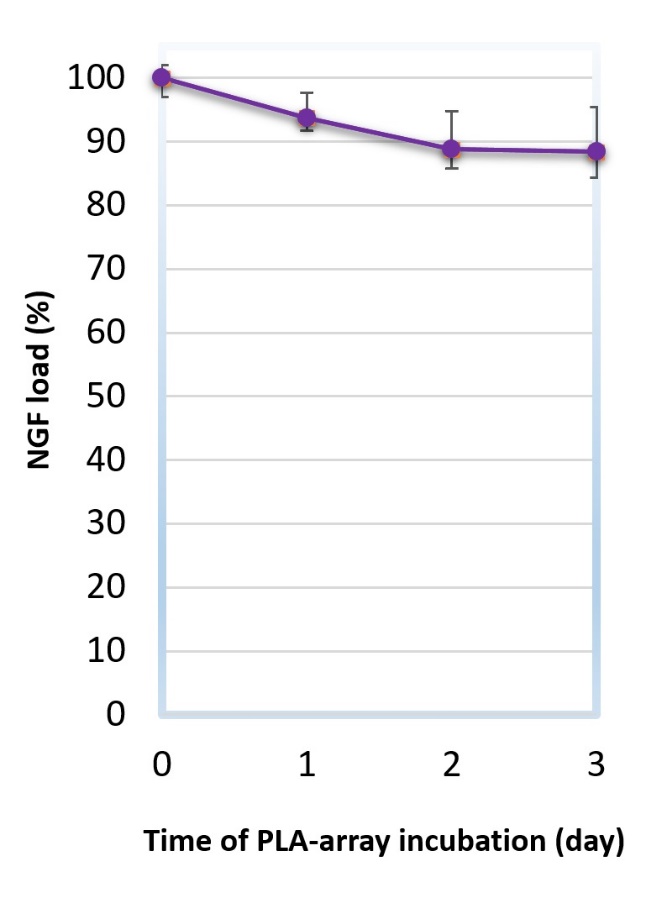


**Supplementary Figure 1.** Time-dependent changes in NGF payload inside PLA-based microchambers. The PLA matrixes were incubated in a Dulbecco's phosphate-buffered saline (DPBS) at 37 ^0^C (95% O_2_, 5% CO_2_) and the amount of NGF was tested in DPBS at different time-points of incubation of the matrixes using spectrophotometry (n = 5 arrays / independent preparations). Average NGF load inside PLA-microchambers is plotted over the time of MCA incubation, reflecting the dynamics of spontaneous NGF release from the microchambers


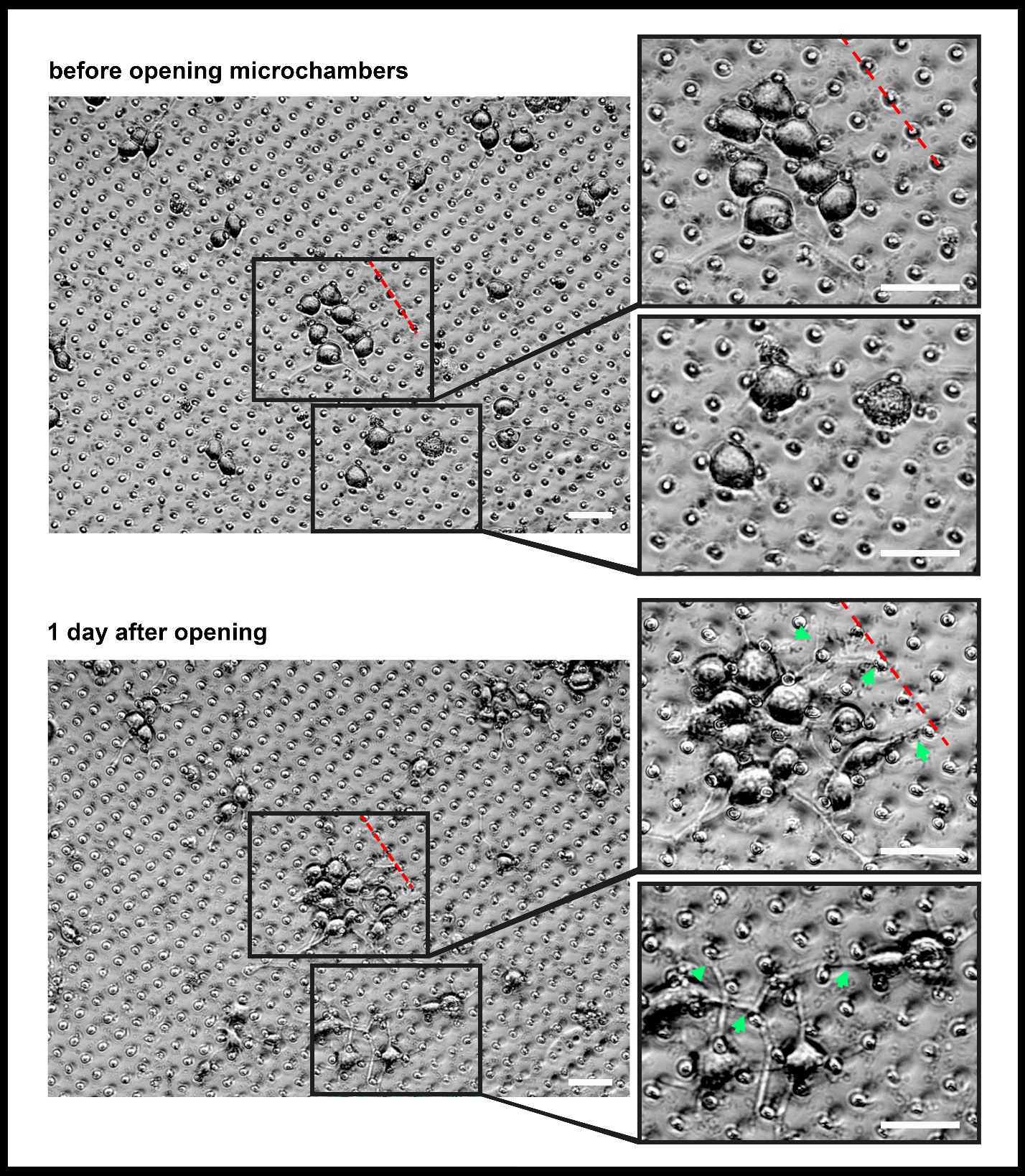


**Supplementary Figure 2.** Neurite outgrowth by local N2A cells towards the triggered NGF release from PLA-microchambers with payload inside. Representative snapshots of differentiating N2A cells on the surface of PLA-based microchambers with NGF inside before microchamber opening (top images) and 1 day after (bottom). Red dotted line, a line segment trajectory for optical targeting microchambers (5 microchambers opened); black boxes, enlargement for the observation of local cells. Note cell neurites directed towards opened depots as marked by green arrows. Scale bar is 40 µm.

**Supplementary Movie 1.** NGF crystallization on the patterned PLA film.

**Supplementary Movie 2.**  N2A cell activity before and following laser-triggered sequential opening of PLA-microchambers loaded with NGF inside. Cell activity (color-coded) was analyzed using the microparticle image velocimetry method and represented as a normalized velocity (blue is minimal movement, red is maximal). Red circles mark the opened microchambers; time of recording indicated; scale bar is 40 µm.

**Supplementary Movie 3.** N2A cell neurite outgrowth directed towards the opened michrochambers (triggered NGF release) shown for a representative cell as in movie 2 for the indicated time period after laser-triggered opening of PLA-microchambers. Notations same as in video 2. Scale bar is 40 µm.
